# Editorial: HTLV-1: addressing unmet research needs, volume II

**DOI:** 10.3389/fmicb.2023.1306416

**Published:** 2023-10-26

**Authors:** Marcela S. Cunha, Wei Zhang, Louis M. Mansky, Luiza M. Mendonça

**Affiliations:** ^1^Department of Biochemistry, Molecular Biology and Biophysics, University of Minnesota, Minneapolis, MN, United States; ^2^Institute for Molecular Virology, University of Minnesota, Minneapolis, MN, United States; ^3^Department of Diagnostic and Biological Sciences, University of Minnesota, Minneapolis, MN, United States

**Keywords:** HTLV, retrovirus, transformation, ATL, HAM/TSP, HBZ

Human T-lymphotropic viruses (HTLVs) are oncogenic human retroviruses. HTLV-1, as the etiological agent of adult T-cell leukemia/lymphoma (ATL), a rare but highly aggressive CD4^+^ T-cell neoplasia, has the most clinical importance. HTLV-1 is associated with other inflammatory disorders such as HTLV-1-associated myelopathy/tropical spastic paraparesis (HAM/TSP), pulmonary disorders, infective dermatitis, and uveitis. Prevalence studies are lacking, but limited data suggest that at least 10 million people are infected, and the actual number is likely closer to 15–20 million infected individuals. Japan and the Caribbean are two areas globally where prevalence rates are significantly higher. HTLV transmission occurs by vertical transmission (predominantly via breastmilk) or horizontally through sexual intercourse, blood transfusions, or shared syringes.

The current therapeutics for HTLV-associated diseases has very limited benefits. Therefore, the development of new therapies is of crucial importance. More basic, applied, and clinical research is needed in order to develop HTLV vaccines and novel treatment options against ATL and HAM/TSP. This Research Topic aims to present the recent discoveries related to potential novel drugs for HTLV-1-related diseases, new research on the link between antisense proteins and diseases, as well as clinical updates on how HTLV-1 infection affects pregnancy outcomes.

Komatsu et al. examined the clinical data of HTLV-1-infected and seroconverted pregnant mothers who enlisted in the database of a prefecture-wide antenatal adult T-cell leukemia prevention program from 2011 to 2018 in Nagasaki, Japan. They produced the first report with chronological data on HTLV-1 proviral load on this population. While infection had no adverse effects on pregnancy outcomes, a positive correlation between the increase of proviral load and the number of parities in HTLV-1 positive women was observed. This report underscores the importance of thorough monitoring for HTLV-1 positive mothers and infants.

Ernzen et al. evaluated the potential of an inhibitor of the protein arginine methyltransferase 5 (PRMT5) to hinder ATL induction. PRMT5 is involved in epigenetics regulation and is overexpressed in HTLV-1-transformed cell lines. They demonstrated that the PRMT5-inhibitor EPZ015666 could prevent the HTLV-1-driven immortalization of cells and exhibited selective toxicity in HTLV-1 transformed cell lines as well as in HTLV-1-infected primary T-cells in a dose- and time-dependent manner. Moreover, the treatment employing the PRMT5-inhibitor significantly reduced the mortality rate in murine models. The authors conclude that PRMT5 is implicated in the HTLV-1-mediated T-cell transformation and disease pathogenesis *in vivo* and that EPZ015666 may have therapeutic value for the treatment of ATL, highlighting the importance of PRMT5 as a potential new target to the development of anti-ATL drugs.

Another study aimed to address the therapeutical potential of histone methyl transferase inhibitors in HTLV-1-associated myelopathy (HAM). Koseki et al. tested if EZH1/EZH2 (enhancer of zeste homolog 1/2) inhibitors could reduce cell proliferation and HTLV-1 proviral load and modulate cytokine production. They concluded that EZH1/2 dual inhibitors can reduce the proliferation of HTLV-1-infected cells and HAM-derived peripheral blood mononuclear cells (PBMC) in a dose-dependent fashion. Additionally, the drugs could suppress the exacerbated immune response in infected cells, as observed by the increase of IL-10, although the levels of IFN-gamma and TNF-alfa were not reduced. These data indicates that the EZH1/2 dual inhibitors may be effective therapeutic agents for HAM. To further confirm these promising data, the authors suggest the conduction of proof-of-concept clinical trials in patients with HAM.

Several retroviruses share an intriguing feature: they have an open reading frame on the negative strand of their genomes, which results in natural antisense transcripts. Lin et al. summarized the main recent findings regarding the antisense transcripts of HTLV-1, HTLV-2, and HIV-1, shedding light on the role of these transcripts and proteins on viral lifecycle, and also on the cellular factors that regulate their transcription. They highlighted that antisense mRNAs of HTLV-1, HTLV-2, and HIV-1 are transcribed from 3' LTR and have multiple transcription start sites. All of them have their expression driven by TATA-less promoters and these antisense transcripts can give rise to proteins. This review also called attention to the role of myocyte enhancer factor-2 (MEF-2) protein family on the regulation of *HBZ* (HTLV-1 basic leucine zipper factor) transcription. The authors also provided an overview of the role of HBZ in ATL disorder: HBZ has several host interacting partners involved in the promotion of T-cell proliferation, transition of G1/S phase, transcriptional factors, and coactivators (such as p300/CPB) and it is considered an inhibitor of tax-mediated transcription, which is a function also played by its HTLV-2 antisense counterpart, the protein APH-2 (HTLV-2 antisense protein). The authors indicate the importance of further research to elucidate the mechanism of MEF-2 family in HBZ regulation and neoplasia. They also suggest antisense genes may be potential targets for therapeutic interventions. To date, CRISPR silencing of *HBZ* was deleterious for ATL cell lines, xenografts, and patient samples, encouraging further studies *in vivo* models.

Liu et al. investigated the potential interaction between HBZ, the nucleolar proteins nucleophosmin (NPM1/B23) and nucleolin (C23), and their implications on the pathogenesis of ATL. They have proven the interaction between NPM1/B23 with HBZ in the nucleolar region in different cell lines. The authors also observed C23 interaction with HBZ. Interestingly, an isoform of HBZ, sHBZ, can interact with its own s*hbz* mRNA. As both proteins are found in nucleolar regions and interact with each other, they hypothesize that NPM1/B23 may form a complex with HBZ and C23. Additionally, downregulation of NPM1/B23 led to a reduction of sHBZ expression along with an increase of Tax expression. In this work, the authors presented novel interaction partners for HBZ, and suggested that these interactions may be involved in HBZ functions, contributing to ATL development and calling attention to the importance of further studies to understand the outcome of these interactions in the pathogenesis of ATL.

In summary, this Research Topic offers valuable insights into potential targets and promising drugs that could broaden the treatment options for diseases caused by HTLV-1. Additionally, it presents recent discoveries related to seroconversion's effects on pregnancy outcomes, explores new partners of the antisense protein HBZ, and sheds light on its role in ATL pathogenesis. These findings collectively enhance our understanding of the mechanisms underlying HTLV-1-related disorders.

## Author contributions

MC: Writing—original draft, Writing–review & editing. WZ: Writing—review & editing. LoM: Writing—review & editing. LuM: Writing—original draft, Writing—review & editing.

